# The value of Morbidity & Mortality (M&M) conferences in residency training: a proposed model from an academic medical center in Iran

**DOI:** 10.1186/s13037-020-0231-3

**Published:** 2020-01-27

**Authors:** Kiana Hassanpour, Nazanin Behnaz, Maryam Fakhri, Mohammad Pakravan

**Affiliations:** Ophthalmic Research Center, Shahid Beheshti University of Medical Sciences, Boostan 9 St. Pasdaran Ave., Tehran, 16666 Iran

Dear Editor;

To increase patient safety, it is the direct responsibility of all healthcare providers to migrate from negligence and approach how to prevent mistakes [1]. In developing and transitional countries, lack of knowledge and responsibility are not always the causative factors leading to patient harm [2].

Mortality and Morbidity conferences (M&M) focusing on learning from errors are required educational series in all residency programs [3]. Based on six core competencies for residents recognized by outcome project of the Accreditation Council for Graduate Medical Education (ACGME) [4], M&M can provide a unique framework to improve these competencies [5]. Among these core competencies, improving systems-based practice as well as interpersonal and communication skills among healthcare providers may result in bridging the wide gap in health care delivery in developing countries. Improving these competencies should be started through the early years of medical education [6].

Working effectively in different health care delivery situations, awareness of cost and risk-benefit analysis, working in inter-professional teams and actively recognizing system errors and applying systems solutions are parts of systems-based practice. Accordingly, Interpersonal and communication skills need the ability to successfully communicate with physicians, other healthcare professionals, and health-related organizations [4].

Morbidity & Mortality conferences, which are described in different methods [7–9], are an imperative element of the residency program. We think they are not sufficiently appreciated in the Ophthalmology residency program which could partially be due to the elective nature of most surgeries in this specialty; and moreover, it is not a life-threatening field of practice. Herein, we report a simulation of M&M in the ophthalmology department at Shahid Beheshti University of Medical Sciences through a real case study. The method applied to hold this session is root cause analysis (RCA). Although this method has been used in other clinical disciplines, we could not find a comparable approach in ophthalmology fields [10, 11]. Furthermore, this is the method suggested by ACGME in simulated patient safety activities [12].

The selected case was a 62-year-old otherwise healthy woman who had undergone uneventful phacoemulsification with one-piece intraocular lens implantation in the right eye 2 weeks earlier. She was not satisfied with the operation and complained of blurry vision afterward. Her refraction before surgery had been − 1.00 D and − 1.50 D for right and left eye, respectively. After the operation, uncorrected distance visual acuity (UDVA) in the right eye was 20/40 which improved to 20/20 with + 2 diopter spectacle correction. The anterior segment, intraocular pressures, optic nerve, and fundus examinations were all unremarkable. Macular optical coherence tomography was normal as well. The induced refractive surprise was attributed to the possible biometry error. The case selected to be scrutinized by further analysis of other possible sources of errors and was referred to the morbidity & mortality committee. This committee’s members consisted of the residents involved, the engaged attending physicians, the head of the optometry department, the optometrist measured the biometry and the operating room staff. The causative factors recognized in the session are shown as a fishbone diagram. (Fig. 1) The key problem was identified to be the lack of careful supervision on novice optometrists who performed biometry, highlighting the fact that they had to be supervised and trained until the learning period was completed. After classifying the problems bellow the subheadings of the patient, people, procedure, environment, equipment and organization and other contributing factors were identified as:

- The inattention of operating resident to the level of expertise of the optometrist who provides the biometric data required for surgery.
- Crowded environments in optometry division and operating room.
- Shortage of required equipment including optical biometry which enhances the accuracy of the test and paucity of the health workforce population in relation to the patient.
- Lack of cooperation of patients during ultrasound biometry.

**Fig. 1:**
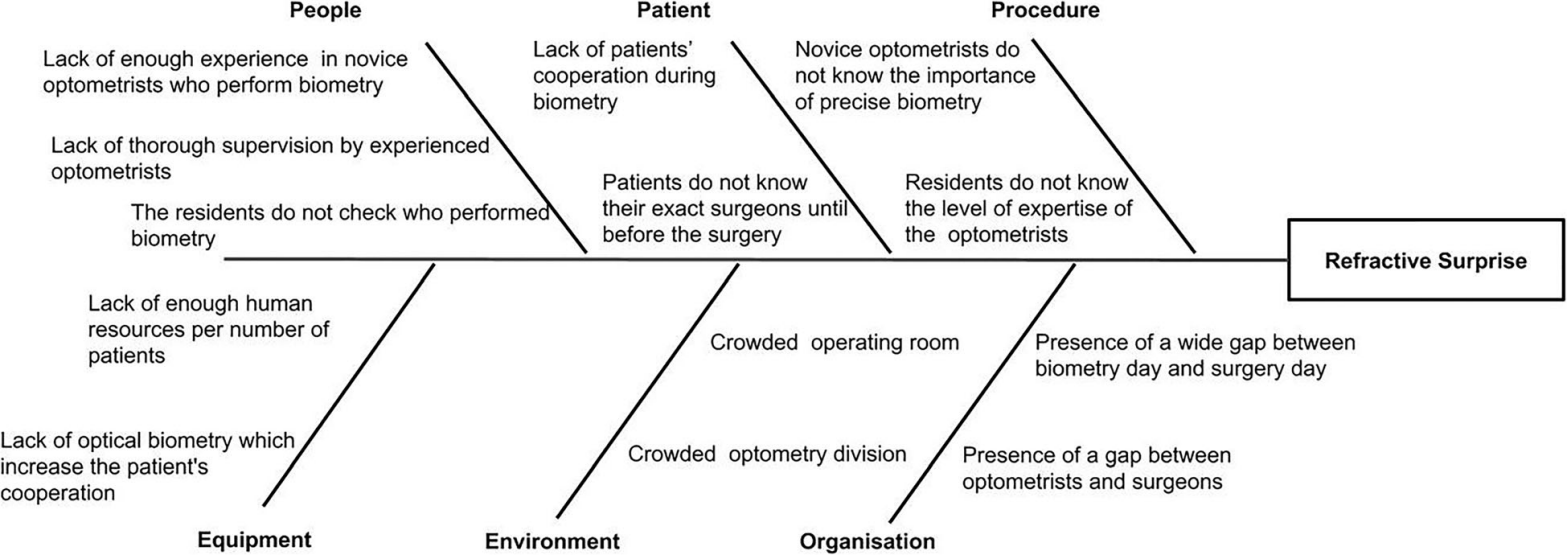
A fishbone diagram showing the root causes of the adverse event which is a refractive surprise

To implement the change, we asked the optometrists to set a new policy for optometry division. The A-scan forms should be signed by the experienced technicians as a sign of supervision. The surgeons should know the level of expertise of the personnel of the optometry division and it is their direct responsibility to confirm the validity of the A-scans.

The benefits of running M&M using this method of analysis include:

- Providing a sincere atmosphere in which the main focus is on finding the root causes of error rather than blaming the individuals.
- Increasing the ability of intercommunication skills among residents and the team who are critical for the excellence of outcome.
- Practicing systems-based thinking through knowing all procedures involved in the context of the working area.

In conclusion, we recommend that holding Morbidity and Mortality conferences regularly using the root cause analysis method is crucial to strengthen patient safety and to increase the residents’ intercomminucation skills and systems-based thinking. These conferences are effective methods to provide the unique opportunity of learning from mistakes in a sincere atmosphere in which the main focus is on the recognition of the root causes of error and prevention of them in the future.

## Data Availability

Not applicable.

## Abbreviations

ACGME: Accreditation Council for Graduate Medical Education
CDVA: Corrected distance visual acuity
M&M: Mortality and morbidity conference
UDVA: Uncorrected distance visual acuity
